# Characteristic rhizosphere metabolites of low-cadmium-accumulating rice drive microbially induced soil cadmium speciation transformation

**DOI:** 10.3389/fmicb.2026.1832350

**Published:** 2026-04-22

**Authors:** Junyang Zhao, Yuhang Qiu, Xiaowen Feng, Guanchun Qin, Shunpiao Meng, Yongzhi Chen, Liyu Shi, Guoyuan Li, Yongcheng Ma, Bing He, Ronghui Wen

**Affiliations:** 1 College of Life Science and Technology/Guangxi Key Laboratory of Agro-Environment and Agric-Products Safety, Guangxi University, Nanning, Guangxi, China; 2 College of Agriculture, Guangxi University, Nanning, Guangxi, China

**Keywords:** cadmium contamination, rhizosphere metabolites, rhizosphere microecology, rhizosphere microorganisms, rice cultivars

## Abstract

**Introduction:**

Cadmium (Cd) contamination in paddy soils threatens global rice safety. However, the mechanisms by which interactions between rhizosphere metabolites and microorganisms regulate Cd bioavailability in the rhizosphere microecosystem of low-Cd-accumulating rice (LAR) remain unclear.

**Methods:**

To elucidate the mechanisms by which metabolite-microbe interactions in the rhizosphere microenvironment of LAR regulate Cd bioavailability, a microplot experiment was performed to classify fifteen rice cultivars into LAR (*n* = 4) and high-Cd-accumulating rice (HAR, *n* = 3) groups.

**Results:**

Rhizosphere omics analyses revealed that LAR tends to form a highly specialized metabolite profile and microbial community at the cost of overall diversity, thereby promoting Cd immobilization. Multiomics integrated analysis demonstrated that characteristic LAR metabolites (Compared with HAR, the rhizosphere metabolites significantly enriched in LAR) serve as carbon sources to enrich Cd-immobilizing bacteria (*Desulfopila aestuarii* and *Paludibacter* sp.) while inhibiting Cd-mobilizing bacteria (*Sulfuriferula* sp. AH1, *Nocardioides deserti*, and *Nocardioides glacieisoli*) through antimicrobial activity. These interactions establish a microecological mechanism that suppresses Cd mobilization and enhances immobilization. Moreover, LAR metabolites increase the abundance of microbial genes encoding enzymes for sinapic acid and biotin biosynthesis-including caffeoyl-CoA O-methyltransferase (EC: 2.1.1.104) and 6-carboxyhexanoate–CoA ligase (EC: 6.2.1.14)-and promote the enrichment of related functional microorganisms such as Candidatus Sulfobium mesophilum and Deltaproteobacteria bacterium, thereby further regulating Cd speciation. Validation experiments revealed that a consortium of 23 characteristic LAR metabolites (e.g., sinapic acid, biotin, isosteviol, hydroxyisocaproic acid, 2-hydroxyhexanoic acid, lumichrome, and hypoxanthine) reduced exchangeable Cd by 10-21% and increased Fe-Mn oxide-bound Cd by 28-56% in both natural and sterilized soils.

**Conclusion:**

These findings reveal that characteristic LAR metabolites directly promote Cd immobilization and drive directional microbial assembly and functional optimization, thereby providing novel insights that can facilitate the establishment of a rhizosphere ecological barrier that can increase resistance to Cd stress in rice.

## 1 Introduction

Cadmium (Cd) is a toxic, nonessential transition metal that poses severe risks to human health (Genchi et al., 2020). According to the (Bulletin of the National Soil Pollution Survey 2014), among the eight major inorganic pollutants, Cd exhibits the highest exceedance rate (7.0%). Among all soil types, farmland has the greatest percentage of Cd-contaminated sites (19.4%), indicating severe levels of Cd pollution in agricultural soils. Compared with other cereals, rice (*Oryza sativa* L.), one of the most important staple crops in China and worldwide, has a relatively strong capacity to accumulate Cd, thus posing severe health risks to humans (Sui et al., 2018; Phillips et al., 2024). Consequently, remediating Cd-contaminated agricultural soils and reducing Cd accumulation in crops have become urgent priorities.

The selection and breeding of LAR constitute effective strategies to mitigate Cd accumulation in rice (Qi et al., 2020). Differences in Cd uptake and accumulation among rice cultivars are governed by multiple mechanisms, with most studies focusing on internal transport processes—for instance, the inhibition of Cd translocation from roots to grains via specific transporters (Lu et al., 2019). Extensive studies have confirmed that *OsNRAMP5* is one of the key transmembrane transporters for Cd absorption by rice roots, and alterations in its expression can significantly influence Cd uptake capacity, thus forming a “high absorption type/low absorption type” variety difference Sasaki et al., 2012; Yang et al., 2014). Yan et al. (2016)) found that *OsHMA3* mediates the sequestration and retention of Cd into root vacuoles, and its enhanced function can reduce Cd translocation to the shoots and consequently decreases Cd accumulation in grains; In contrast, genotypes with defective OsHMA3 function tend to exhibit a high-Cd-accumulating phenotype. Sasaki et al. (2014)) further demonstrated that the overexpression of functional *OsHMA3* in model Japonica Rice Varieties reduced the translocation and accumulation of Cd in grains. However, the understanding of how interactions between rhizosphere metabolites and microorganisms in the rhizosphere microecosystems of different types of rice influence Cd bioavailability remains limited.

The rhizosphere—the soil region adjacent to plant roots—is among the most complex and ecologically active interfaces in plant–soil systems (Wang et al., 2023). Within this zone, the intricate interactions between metabolites and microorganisms not only participate in plant nutrient acquisition but also substantially influence the bioavailability of heavy metals (Wen et al., 2023). Rhizosphere metabolites, comprising primary and secondary compounds released by plants and associated microbes (Cheng et al., 2022), can directly reduce Cd bioavailability through chelation and complexation. For instance, 6,7-diethoxy-4-methylcoumarin and poly-γ-glutamic acid effectively stabilize Cd in soils (Szwaczko et al., 2025; Wang X. et al., 2020). In addition to direct chemical effects, rhizosphere metabolites play pivotal roles in recruiting and driving microbial communities (Sasse et al., 2018; Huang et al., 2019; Wen et al., 2022). Rhizosphere microorganisms can activate or stabilize soil Cd through chemical transformation, chelation, protonation, precipitation, and biosorption (Song et al., 2018). Thus, metabolites act as rhizosphere signaling molecules that recruit specific microbial taxa, thereby markedly altering the microbial community composition and consequently influencing heavy metal bioavailability (Yue et al., 2023; Zhou et al., 2023). For example, under heavy metal stress, neocnidilide secreted by *Miscanthus* promotes the growth of *Glomeromycota*, thereby increasing the release of polysaccharide-like colloidal substances that adsorb heavy metals, thus reducing their bioavailability (Wu et al., 2024). Plant species and even cultivars present distinct rhizosphere microecosystems that substantially influence Cd accumulation in crops (Zhang et al., 2022). Li et al. (2023)) demonstrated that variations in rhizosphere bacterial communities among rice cultivars contribute to differences in Cd and mineral element accumulation in brown rice. Similarly, Wu et al. (2023)) reported that under Cd stress, differential rhizosphere metabolites in LAR and HAR rapeseed cultivars (L338 and L351, respectively) selectively recruited distinct microbial groups, leading to differences in Cd uptake. Therefore, we hypothesize that different rice cultivars harbor unique rhizosphere microecosystems in which distinct rhizosphere metabolites drive the assembly of characteristic microbial communities that ultimately influence Cd speciation transformation.

However, the mechanisms through which rhizosphere metabolites drive microbial community assembly in LAR under Cd-contaminated conditions remain unclear. In this study, a microplot experiment involving fifteen rice cultivars was conducted in Cd-contaminated soils. First, we compared Cd accumulation in rice and Cd speciation in the rhizosphere soils among different cultivars to identify the distinct uptake and accumulation characteristics of LAR and HAR. Second, an integrated approach combining metabolomics and metagenomics was employed to identify key rhizosphere metabolites potentially influencing Cd bioavailability and to elucidate the underlying metabolite–microbe interactions within the rhizosphere microecosystems. Finally, differentially abundant metabolites identified from LAR were formulated into preparations and tested in soil incubation experiments to provide direct evidence of metabolite-driven Cd speciation transformation. This study aims to provide new insights that can help mitigate the toxicity of Cd in soil and establishing a primary ecological barrier to increase rice resistance to Cd stress, thereby contributing to the sustainable and safe production of rice.

## 2 Materials and methods

### 2.1 Microplot experiment

**Figure 1:**
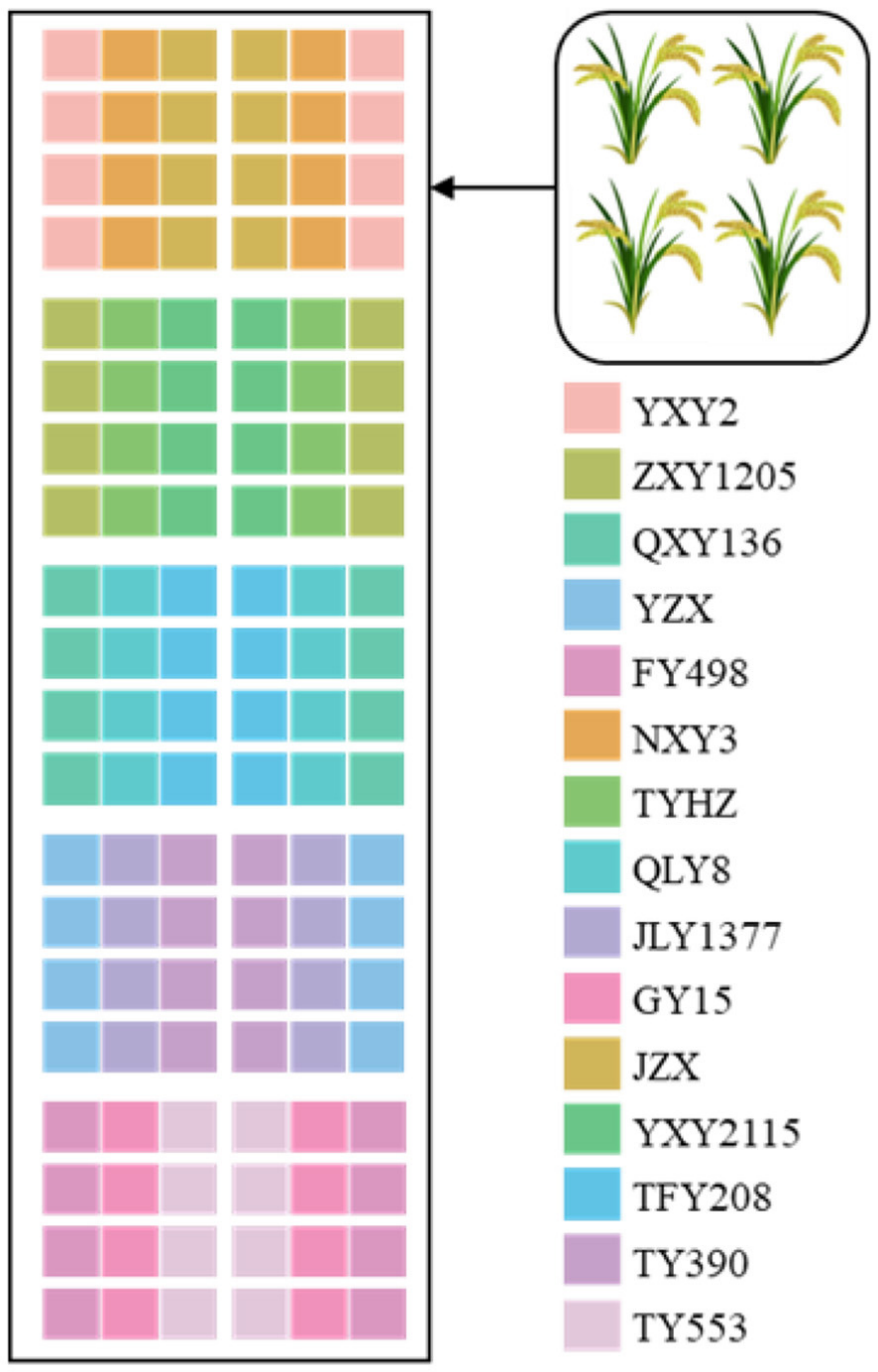
Schematic diagram of micro-plot experimental design for rice variety screening.

A microplot experiment was conducted in 2023 at the research base of Guangxi University, Nanning City, Guangxi Zhuang Autonomous Region, China. The soil tanks used for the experiments measured 4.8 m (length) × 1.2 m (width) × 0.4 m (height). Soil was collected from the top 0–20 cm layer of a Cd-contaminated paddy field in Qintang District, Guigang City, Guangxi Zhuang Autonomous Region, China. The collected soil was air-dried, ground and sieved through 20 mm mesh. The detailed physicochemical properties of the soil are summarized in Supplementary Table S1. Fifteen rice cultivars (Supplementary Table S2) were selected, and plump seeds of each were surface-sterilized, soaked, and germinated. After 25 days of seedling cultivation in a growth substrate, uniform seedlings were transplanted into the experimental tanks on 31 July 2023. Each cultivar was planted in eight clumps, with two seedlings per clump. To minimize root interference among cultivars, each variety was planted separately in root bags (17 × 20 cm). After the root bags were placed in the field soil, rigid plastic isolation panels were installed between adjacent varieties, extending 30 cm below the soil surface, with a 15 cm buffer zone maintained on both sides of each panel to effectively block horizontal root extension (Figure 1). The planting density was maintained at 20 × 25 cm (row × hill spacing). All plots were managed uniformly to ensure consistent growth conditions and reliable experimental results. Fertilization was performed according to local conventional practices. A compound fertilizer (N:P_2_O_5_:K_2_O = 15:15:15) was used as the basal fertilizer at 375 kg ha^−1^, and urea was employed as a topdressing at 112.5 kg ha^−1^ during the tillering stage on 20 August 2023.

### 2.2 Soil and plant sample collection

Rhizosphere soil samples were collected during the filling stage on 11 October 2023. The rhizosphere soil was prepared as follows: loosely adhering soil was gently shaken off, and approximately 1 mm of soil was left tightly attached to the root surface. The remaining soil adhering to the roots was then carefully washed off using sterile water. The resulting soil suspension was divided into two portions: one portion was freeze-dried for rhizosphere metabolomic analysis, and the other was centrifuged at 10,000 × g for 10 min. The pellet was divided into two subsamples—one for DNA extraction and the other for Cd speciation. At the rice harvest stage on 1 November 2023, root and grain samples were collected. All samples were heated to 105 °C for 1 h and dried at 65 °C until they reached constant weights. The dry samples were then ground, passed through a 60 mm mesh sieve, and stored at 25 °C.

### 2.3 Soil Cd speciation

Soil Cd speciation was determined using the Tessier sequential extraction method (Tessier et al., 1979). The detailed extraction procedures and experimental conditions are presented in Supplementary Table S3. Each fraction was passed through a 0.45-μm filter membrane and examined using a graphite furnace atomic absorption spectrophotometer (PinAAcle 900T, PerkinElmer, Waltham, MA, United States).

### 2.4 Determination of Cd content in roots and grains

Rice samples (0.2000 g, roots and grains) were digested in concentrated nitric acid (98%) using a microwave digestion instrument (MARS, CEM company, Matthews, North Carolina). The Cd concentration was then determined by inductively coupled plasma mass spectrometry (ICP–MS) (NexION 2000, PerkinElmer). Quality control was ensured by using the certified reference material GBW100348 (plant matrix) provided by the National Research Center for Standard Materials (China). The three rice cultivars with the highest Cd concentrations in roots and grains were classified as the HAR, whereas the four cultivars with the lowest Cd concentrations were designated as the LAR. Roots, grains, and rhizosphere soils from the same Cd accumulation type (low or high) were composited separately as biological replicates. This composite sampling strategy was employed to capture representative characteristics more effectively and to facilitate comparative analyses among rice types with distinct Cd accumulation capacities.

### 2.5 Rhizosphere soil metabolite extraction and analysis

To identify rhizosphere metabolites that potentially drive the assembly of microbial communities in LAR, metabolites were extracted from the rhizosphere soils of the LAR and HAR groups and analyzed following a modified version of the protocol described by Wen et al. (2023)).

Approximately 100 ± 1 mg of soil was placed in a tube containing beads and mixed with 500 μL of extraction solvent (methanol: acetonitrile: water, 2:2:1, v/v/v) containing deuterated internal standards. The mixture was vortexed for 30 s, homogenized (35 Hz, 4 min) and sonicated for 5 min in a 4 °C water bath. This homogenization–sonication process was repeated three times to ensure complete extraction. The samples were then incubated at −40 °C for 1 h to precipitate the proteins, which were subsequently centrifuged at 12,000 rpm (≈13,800 × g, *R* = 8.6 cm) for 15 min at 4 °C. The resulting supernatant was transferred to a clean glass vial for liquid chromatography–tandem mass spectrometry (LC–MS/MS) analysis. Quality control (QC) samples were prepared by pooling equal aliquots from all the supernatants.

LC–MS/MS analyses were performed on an ultrahigh-performance liquid chromatography (UHPLC) system (Vanquish, Thermo Fisher Scientific, Wilmington, DE, United States) equipped with a Phenomenex Kinetex C18 column (2.1 × 50 mm, 2.6 μm) coupled to an Orbitrap Exploris 120 mass spectrometer (Thermo Fisher Scientific). The mobile phases consisted of (A) 0.01% acetic acid in water and (B) isopropanol–acetonitrile (1:1, v/v). The autosampler temperature was maintained at 4 °C, with an injection volume of 2 μL. The Orbitrap Exploris 120 was operated in information-dependent acquisition (IDA) mode using Xcalibur software (Thermo Fisher Scientific). In this mode, the software continuously evaluates full-scan MS spectra to trigger MS/MS acquisition. The electrospray ionization (ESI) source parameters were as follows: sheath gas flow rate, 50 Arb; auxiliary gas flow rate, 15 Arb; capillary temperature, 320 °C; spray voltage, 3.8 kV (positive mode) or −3.4 kV (negative mode); full MS resolution, 60,000; MS/MS resolution, 15,000; and stepped normalized collision energy (NCE), 20, 30, and 40.

### 2.6 Rhizosphere microbiome sequencing and analysis

Total genomic DNA of soil microorganisms in each sample was extracted in triplicate using the E.Z.N.A.^®^ Soil DNA Kit (Omega Bio-Tek, Norcross, GA, United States) following the manufacturer's instructions. The concentration and purity of the extracted DNA were measured using a NanoDrop 2000 spectrophotometer (Thermo Fisher Scientific), and DNA integrity was assessed by 1% agarose gel electrophoresis. High-quality DNA samples were stored at −80 °C for subsequent metagenomic sequencing on the Illumina HiSeq 2500 platform (Meige Gene Co., Ltd., Guangdong, China).

Quality control of the raw sequencing reads was performed with the assistance of Fastp (v0.23.2) to remove adapter sequences and low-quality reads (Chen, 2023). Taxonomic classification of the clean reads was conducted using Kraken2 (v2.1.3), and the relative abundance was refined using Bracken (v2.9) (Lu et al., 2022). Metagenomic assembly was performed with MEGAHIT (v1.2.9), and open reading frames (ORFs) were predicted using Prodigal (v2.6.3) (Li et al., 2016). To reduce redundancy, non-redundant gene catalogs were constructed using CD-HIT (v4.6) (Fu et al., 2012). Representative sequences from the non-redundant gene catalog were aligned to the NCBI NR database (e-value ≤ 1e−5) using DIAMOND (v0.8.35) (Buchfink et al., 2015) (http://www.diamondsearch.org/) for taxonomic annotation. Functional annotation was performed by aligning the representative sequences against the Kyoto Encyclopedia of Genes and Genomes (KEGG) database (http://www.genome.jp/kegg/) using DIAMOND with an e-value cutoff of 1e−5 (Buchfink et al., 2015).

### 2.7 Network analysis

A network analysis was performed to explore co-occurrence patterns among microbial taxa and functional genes in rhizosphere soils. The 200 most abundant taxa and functional categories were selected for analysis. Spearman's rank correlation coefficients were calculated using the “cor” function in R (v4.1.3), with correlation thresholds of |*r*| > 0.8 and *P* < 0.05, to identify significant microbial co-occurrence relationships. Additionally, co-occurrence networks were constructed to investigate potential interactions between key metabolites and dominant microbial taxa within the LAR and HAR groups. For metabolite–microbe correlations, thresholds of |*r*| > 0.6 and *P* < 0.05 were applied. The topological properties of the networks—including the number of nodes and edges, network density, average clustering coefficient, eigenvector centrality, and modularity coefficient—were calculated using the built-in functions of Gephi (v0.10.1). Network visualization was performed in Gephi to illustrate the complex patterns of interaction among rhizosphere microorganisms and metabolites.

### 2.8 Effects of rhizosphere metabolites on soil Cd speciation

To investigate the direct and indirect effects of rhizosphere metabolites from LAR on reducing bioavailable Cd in soil, a soil validation experiment was conducted in Petri dishes. According to the relative abundance, VIP scores, fold changes, safety for humans and commercial availability for subsequent experimental validation of LAR rhizosphere metabolites (Supplementary Table S19), 23 compounds were selected, including sinapic acid, isosteviol, 6,7-diethoxy-4-methylcoumarin, retinoic acid, dehydroabietic acid, lumichrome, hypoxanthine, allopurinol, hecogenin, imatinib, totarol, hydroxyisocaproic acid, 2-ethyl-2-hydroxybutyric acid, 2-hydroxyhexanoic acid, isoferulic acid, ferulate, arachidonic sulfonic acid, pentadecanal, biotin, undecanoic acid, homoveratric acid, hydroxytyrosol acetate, and hydroferulic acid.

Soil samples were collected from corresponding plots in the microplot experiment. A portion of the soil was sterilized by autoclaving at 121 °C for 20 min on two consecutive occasions, followed by a third round of autoclaving 24 h later to ensure complete sterilization. The absence of cultivable microorganisms in sterilized soils was confirmed by plating soil dilutions on LB agar. The 23 selected metabolites were then prepared as an equimolar mixture (designated as mixture C; 100 μM per compound). The experiments included two treatments under two soil conditions (natural and sterile), each with four replicates: (1) CK: sterile water; and (2) T: metabolite mixture C. In each treatment, 60 g of soil was placed in 9-cm Petri dishes and incubated at 28 °C for 2 weeks in a growth chamber to allow microbial community stabilization. Following stabilization, 10 mL of metabolite mixture C or sterile water was applied weekly for 4 weeks. All Petri dishes were randomly arranged during incubation. Soil moisture was maintained by weighing twice a week and adjusting to 80% of the water holding capacity using sterile deionized water. After 4 weeks, soil from all Petri dishes of each treatment was collected, and Cd speciation was determined to assess changes in soil Cd bioavailability.

### 2.9 Validation of low- and high-Cd-accumulating rice cultivars

To evaluate the stability of Cd uptake and accumulation differences between LAR and HAR, a pot experiment was conducted on April 30, 2024. The selected LAR were NXY3, ZXY1205, YXY2, and YXY2115, while the HAR included GY15, TY390, and TY553. The soil used in the pot experiment was the same as that described in Section 2.1.The experiment was carried out at the research base of Guangxi University using black cylindrical plastic pots (40 × 35 × 30 cm), each filled with 6 kg of soil. Seed soaking, germination, transplanting, and subsequent crop management followed the procedures described in Section 2.1. Rice samples were collected at maturity on August 30, 2024, and Cd concentrations were determined according to the methods described in Sections 2.4.

### 2.10 Data analysis and visualization

A one-way analysis of variance (ANOVA) was performed to assess differences in bioavailable Cd in the soil. Graphical visualizations were generated using Origin 2024. Bioinformatic analyses of rhizosphere metabolomic and metagenomic data were conducted on the MegCloud platform. Partial least squares path modeling (PLS-PM), implemented with the plspm package in R (v4.1.3), was used to evaluate the relationships among soil metabolites, microbial communities, Cd fractions, and rice Cd accumulation. A bootstrap resampling procedure with 1,000 iterations was used to validate the estimates of path coefficients and coefficients of determination (*R*^2^). The goodness-of-fit (GOF) index was subsequently used to assess the overall predictive performance of the model.

## 3 Results

### 3.1 Cd accumulation characteristics of different rice cultivars and Cd speciation in rhizosphere soil

**Figure 2:**
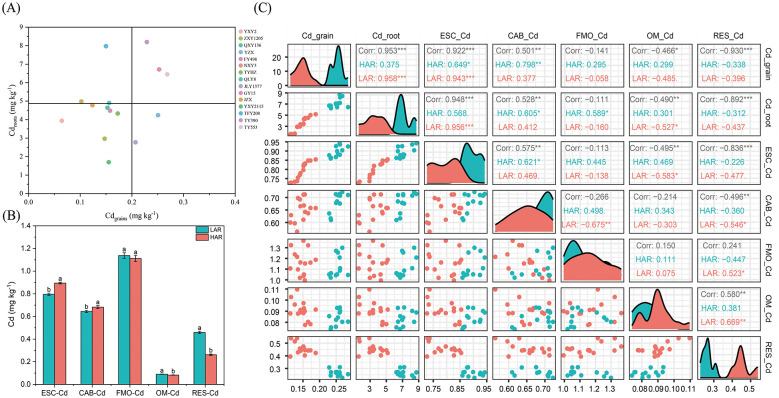
Cd accumulation characteristics of different rice cultivars and chemical speciation of Cd in rhizosphere soils. **(A)** Cd concentration in root and grain of 15 rice cultivars. **(B)** Chemical speciation of Cd in the rhizosphere soils of LAR and HAR. ESC-Cd, CAB-Cd, FMO-Cd, OM-Cd, and RES-Cd represent exchangeable cadmium, carbonate-bound cadmium, iron-manganese oxide-bound cadmium, organic-bound cadmium, and residual state cadmium, respectively. **(C)** Spearman's correlation analysis between Cd speciation and Cd concentrations.

The 2-year cultivar screening revealed clear differences in Cd accumulation among rice cultivars. Based on Cd concentrations in grains and roots, cultivars with relatively low Cd accumulation (NXY3, ZXY1205, YXY2, and YXY2115) were consistently grouped as LAR, whereas cultivars exhibiting higher Cd accumulation (GY15, TY390, and TY553) were classified as HAR (Figure 2A and Supplementary Figure S1). In the rhizosphere soils (Figure 2B), compared with those in HAR soils, the exchangeable cadmium (ESC-Cd) and carbonate-bound cadmium (CAB-Cd) contents in LAR soils were significantly lower (by 11.1 and 5.6%, respectively), whereas the organic-bound cadmium (OM-Cd) and residual state cadmium (RES-Cd) contents significantly increased (*P* < 0.05). Moreover, variations in ESC-Cd, CAB-Cd, OM-Cd, and RES-Cd were significantly correlated with Cd accumulation in rice (Figure 2C). These findings suggested that Cd speciation in the rhizosphere of different rice types plays a decisive role in rice Cd accumulation, indicating that Cd accumulation levels in different rice cultivars are closely linked to the rhizosphere microecological system.

### 3.2 Rhizosphere metabolomic profiles of rice cultivars with differential Cd accumulation

**Figure 3:**
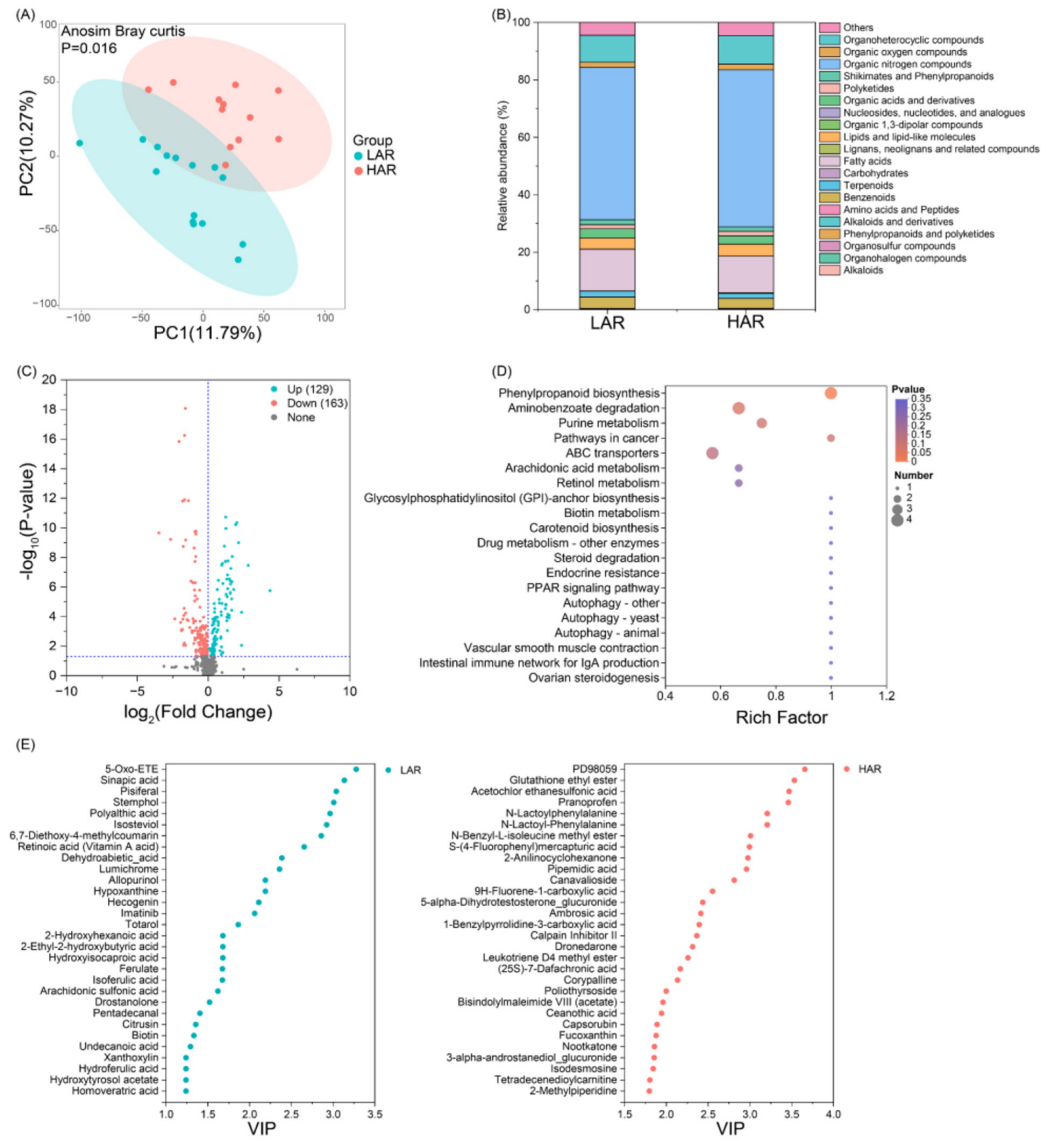
Rhizosphere metabolomic profiles of rice cultivars with differential Cd accumulation. **(A)** Principal component analysis (PCA) of rhizosphere metabolites based on Bray–Curtis distance (*P* < 0.05). **(B)** Stacked bar chart showing the secondary classification (superclass) of rhizosphere metabolites. **(C)** Volcano plot illustrating the differential metabolites between LAR and HAR groups (FC > 1.0 and *P* < 0.05). **(D)** KEGG pathway enrichment analysis of differential rhizosphere metabolites. **(E)** VIP values of differential metabolites in LAR and HAR rhizospheres.

A principal component analysis (PCA) of the rhizosphere soil metabolomes revealed that different rice cultivars have significantly altered rhizosphere metabolite compositions (Figure 3A). A total of 999 metabolites were detected in the rhizosphere soil. Compared with HAR rhizospheres, LAR rhizospheres presented higher relative abundances of organic acids and derivatives, shikimates and phenylpropanoids, benzenoids, terpenoids, and fatty acids (Figure 3B). A total of 129 metabolites, defined as characteristic LAR metabolites, were significantly upregulated in the LAR rhizospheres. These metabolites included 5-oxo-ETE, sinapic acid, isosteviol, and 6,7-diethoxy-4-methylcoumarin, which were markedly enriched and exhibited high variable importance in projection (VIP) scores. Conversely, 163 metabolites were significantly upregulated in HAR rhizospheres and were defined as HAR-specific metabolites (Figure 3C; Supplementary Tables S4, S5). These included PD98059, glutathione ethyl ester, acetochlor ethanesulfonic acid, and pranoprofen, which were highly enriched and had elevated VIP scores (Figure 3E). Further analysis revealed that characteristic LAR metabolites were composed mainly of fatty acids, benzoids, and organic acids and derivatives, with relative abundances of 41.5, 17.0, and 14.1%, respectively. In contrast, characteristic HAR metabolites were more uniformly distributed, with relative abundances of benzoids, lipids and lipid-like molecules, shikimates and phenylpropanoids, and fatty acids accounting for 19.7, 12.9, 11.3, and 10.5%, respectively (Supplementary Table S6). KEGG pathway enrichment analysis of characteristic LAR metabolites revealed that differentially abundant metabolites were predominantly involved in phenylpropanoid biosynthesis (Figure 3D), thus suggesting that increased phenylpropanoid metabolism may play a critical role in modulating Cd bioavailability in LAR rhizospheres.

### 3.3 Rhizosphere microbial community characteristics of rice cultivars with differential Cd accumulation

Metagenomic sequencing was performed on rhizosphere soil samples from LAR (*n* = 15) and HAR (*n* = 12). The sequencing data are summarized in Supplementary Table S7. After quality control of the raw reads was performed, more than 80% of the total sequences were clean reads. The assembled contigs (Supplementary Table S8) were subsequently used for taxonomic and functional annotations.

**Figure 4:**
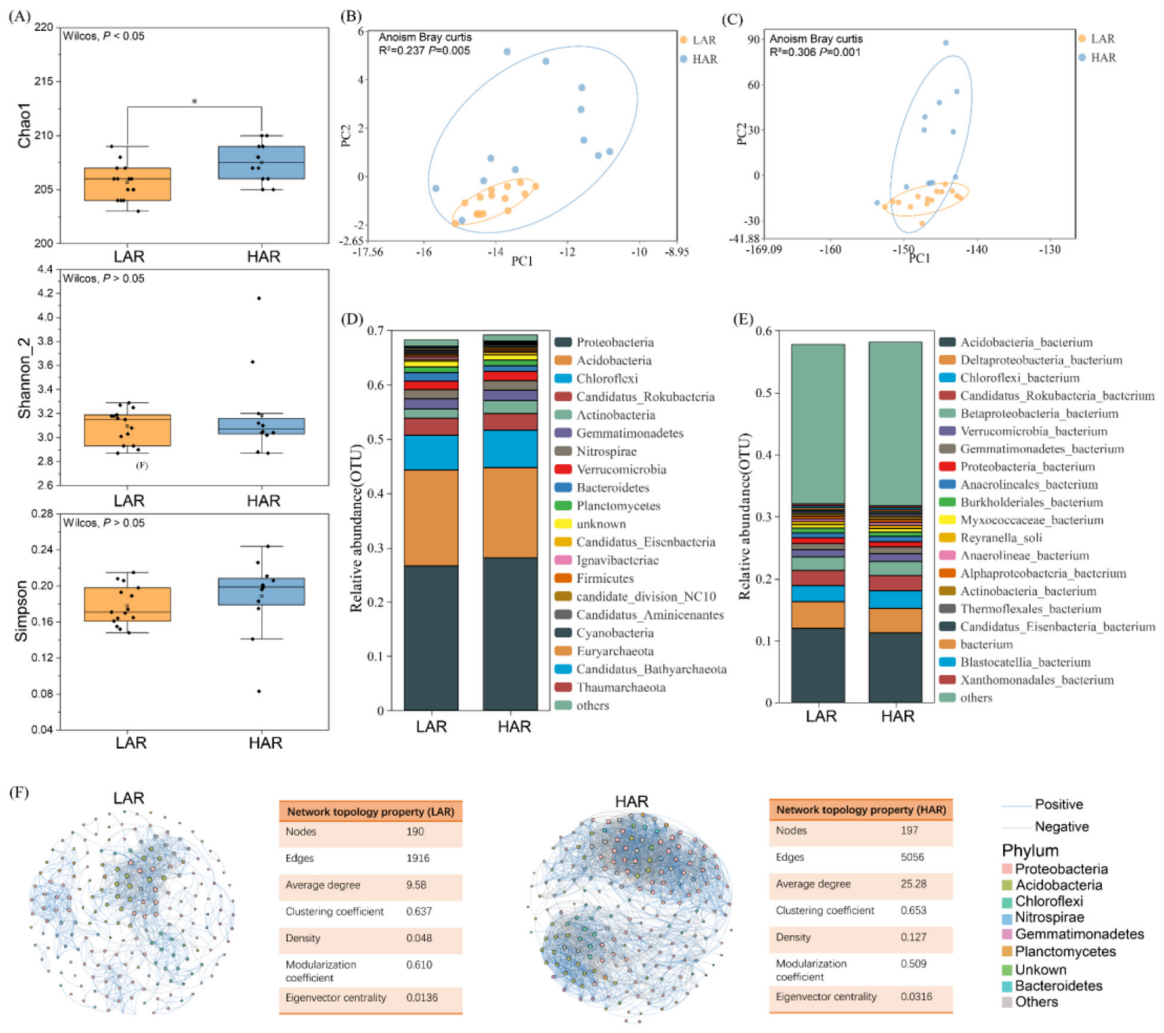
Community composition and diversity of rhizosphere microorganisms in rice cultivars with different Cd accumulation capacities. **(A)** Chao1, Shannon, and Simpson indices of rhizosphere microbial communities. **(B)** Principal component analysis (PCA) of microbial communities at the phylum level based on Bray–Curtis distance. **(C)** PCA of microbial communities at the species level based on Bray–Curtis distance. **(D)** Relative abundance (%) of dominant phylum in the rhizosphere microbial communities. **(E)** Relative abundance (%) of dominant species in the rhizosphere microbial communities. **(F)** Co-occurrence network visualization and topological statistics of rhizosphere microbial interactions. Light blue edges indicate significant positive correlations, and light gray edges indicate significant negative correlations.

Compared with those in the HAR rhizosphere, the Chao1 index in the LAR rhizosphere was significantly lower (*P* < 0.05), whereas the Shannon index slightly increased and the Simpson index slightly decreased (*P* > 0.05) (Figure 4A). On the basis of the results of Bray–Curtis distance and ANOSIM analyses, the bacterial communities in the LAR and HAR rhizospheres were clearly separated at both the phylum (Figure 4B) and species levels (Figure 4C) (*P* < 0.01). These results indicate a pronounced difference in the rhizosphere bacterial community compositions between LAR and HAR. Taxonomic analysis revealed that the rhizosphere microbial communities of both rice types were primarily composed of ten dominant phyla (Figure 4D), namely, Proteobacteria (26.7%−28.1%), followed by Acidobacteria, Chloroflexi, Candidatus Rokubacteria, Verrucomicrobia, Nitrospirae, Bacteroidetes, Gemmatimonadetes, Actinobacteria, and Planctomycetes. Linear discriminant analysis effect size (LEfSe) revealed that Bacteroidetes, Ignavibacteriae, and Euryarchaeota were significantly enriched in LAR, whereas Proteobacteria, Actinobacteria, Verrucomicrobia, and Microsporidia were significantly enriched in HAR (Supplementary Figure S2). At the species level (Figure 4E), the most abundant taxa included *Acidobacteria bacterium* (11.2%−12.0%), *Deltaproteobacteria bacterium* (3.96%−4.28%), *Chloroflexi bacterium, Candidatus Rokubacteria bacterium, Betaproteobacteria bacterium*, and *Verrucomicrobia bacterium*. Kruskal–Wallis analysis (Supplementary Figure S3) revealed that *Deltaproteobacteria bacterium, Blastocatellia bacterium* AA13, and *Bacteroidetes bacterium* were enriched in LAR, whereas *Actinobacteria bacterium* and *Nitrospira* sp. were significantly enriched in HAR (*P* < 0.05).

Network analysis provided integrative insights into microbial community dynamics and revealed co-occurrence patterns at the phylogenetic level. A co-occurrence network based on the 200 most abundant microbial species (|*r*| > 0.8, *P* < 0.05) was constructed (Figure 4F). The LAR rhizosphere network consisted of 190 nodes (species) and 1,916 edges, whereas the HAR network comprised 197 nodes and 5,056 edges. The average network degree was 9.58 for LAR and 25.28 for HAR. Compared with HAR, the LAR network exhibited lower density, clustering coefficient, and eigenvector centrality but a higher modularity coefficient. These results indicate that the rhizosphere microbial network of HAR was larger and more complex than that of LAR, thus reflecting the distinct microbial community structures between the two Cd accumulation types.

### 3.4 Functional analysis of the rhizosphere microbial communities of rice cultivars with differential Cd accumulation

**Figure 5:**
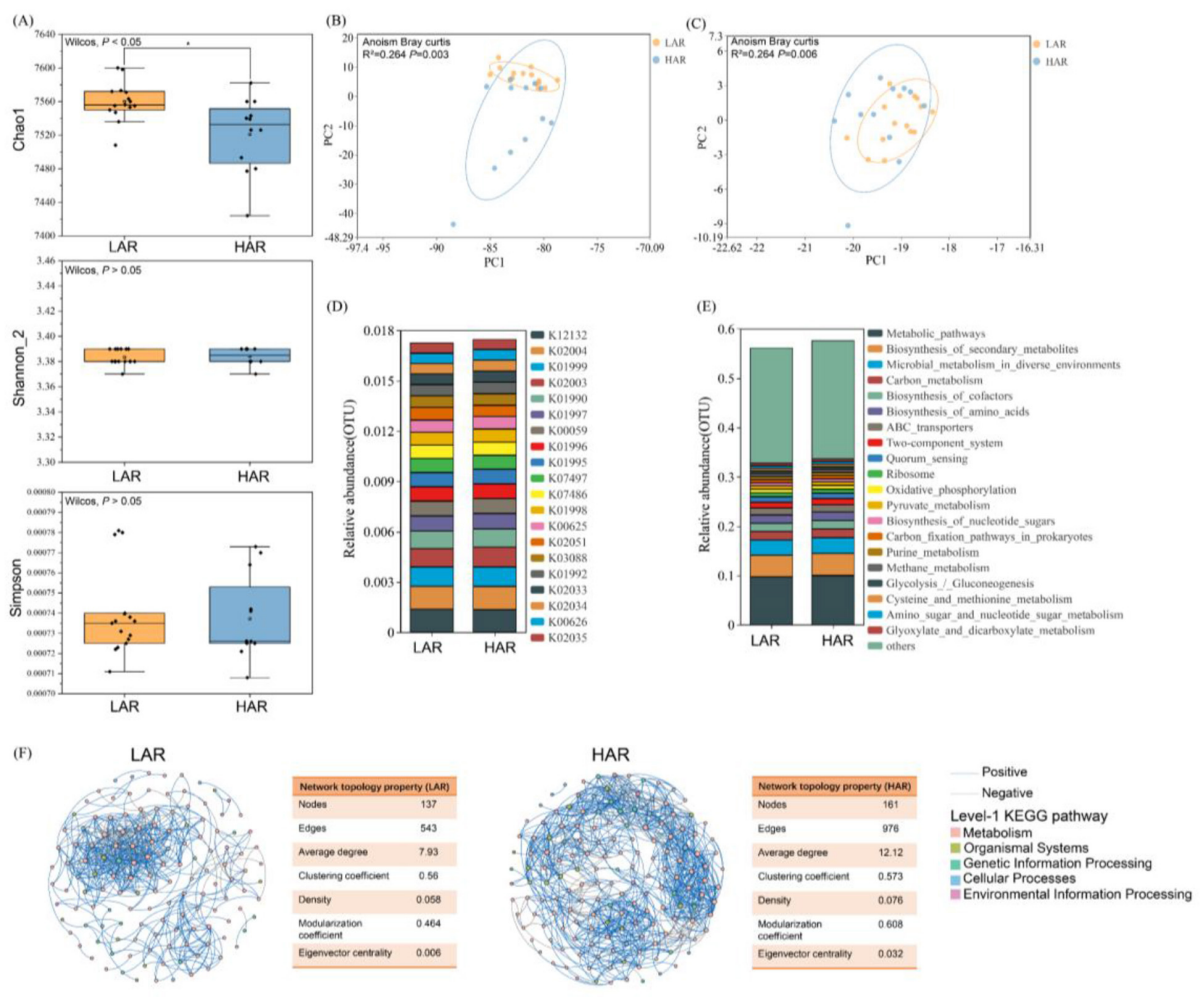
Functional composition and diversity of rhizosphere soil microorganisms in rice cultivars with different Cd accumulation capacities. **(A)** Chao1, Shannon, and Simpson indices of rhizosphere microbial functions. **(B)** PCA plot based on KO. **(C)** PCA plot based on level-3 KEGG functional categories. **(D)** Relative abundance (%) of functions at the KO level. **(E)** Relative abundance (%) of functions at level-3 KEGG categories. **(F)** Co-occurrence network visualization and topological statistics of rhizosphere microbial functions. Light blue lines indicate significant positive correlations, while light gray lines indicate significant negative correlations.

To elucidate the potential ecological functions of rhizosphere microbial communities, we integrated microbial taxonomic profiles with functional annotations based on the KEGG database. A total of 7,680 KEGG Orthologs (KOs) were identified and categorized into 467 level-3 pathways. Compared with those in the HAR rhizosphere, the Chao1 index of microbial functional diversity in the LAR rhizosphere was significantly lower (*P* < 0.05), whereas the Shannon index slightly increased and the Simpson index slightly decreased (*P* > 0.05) (Figure 5A). A PCA and ANOSIM based on the Bray–Curtis distance revealed a clear separation of rhizosphere microbial functions between the LAR and HAR groups at both the KEGG-KO (Figure 5B) and KEGG-L3 (Figure 5C) levels (*P* < 0.01), indicating distinct microbial functional compositions.

A functional composition analysis revealed that the dominant annotated KOs across all the samples included K12132, K02004, K01999, K02003, and K01990 (Figure 5D). Compared with HAR, in which K02003, K01990, K01992, and K02033 were enriched, functions such as K02051 and K07045 were significantly enriched in LAR (Supplementary Table S9). With respect to level-3 KEGG pathway classifications (Figure 5E), the most abundant functions included metabolic pathways (9.76%−10.0%), biosynthesis of secondary metabolites (4.34%−4.48%), microbial metabolism in diverse environments, carbon metabolism, biosynthesis of cofactors, and biosynthesis of amino acids. Kruskal–Wallis analysis (Supplementary Table S10) revealed that signaling pathways (e.g., Wnt, mTOR, Apelin, and cAMP) and biosynthetic processes (e.g., cutin, suberin and wax biosynthesis; biosynthesis of various alkaloids; and flavonoid biosynthesis) were significantly enriched in LAR, whereas metabolic pathways, biosynthesis of secondary metabolites, biosynthesis of amino acids, and biosynthesis of cofactors were significantly enriched in HAR (*P* < 0.05).

A co-occurrence network analysis was performed on the basis of the 200 most abundant level-3 KEGG pathways (|*r*| > 0.8, *P* < 0.05). The LAR functional network consisted of 137 nodes and 543 edges, whereas the HAR network comprised 161 nodes and 976 edges (Figure 5F). Like the microbial community network, the rhizosphere microbial functional network also exhibited pronounced structural differences, and the HAR network was larger and more complex than that of LAR.

### 3.5 Associations between rhizosphere metabolites and microbial communities

**Figure 6:**
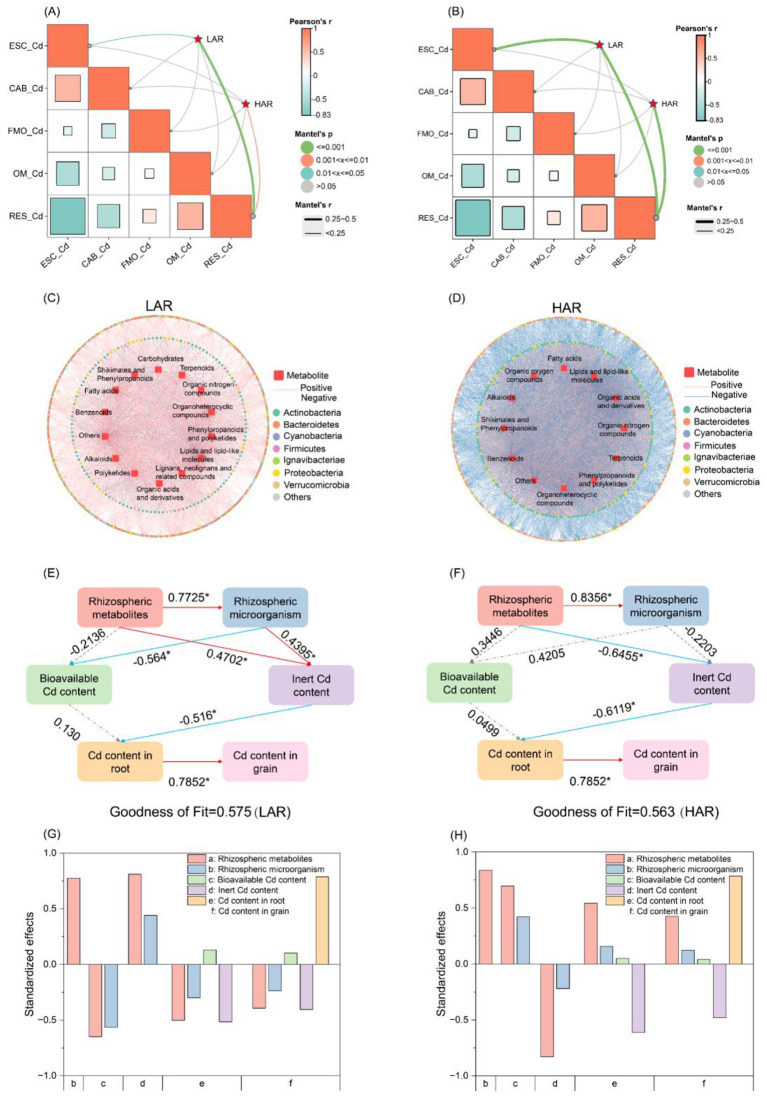
Association analysis of rhizosphere metabolites, microbial communities, and Cd speciation in LAR and HAR. **(A)** Mantel test of bacteria significantly enriched in the LAR and HAR rhizospheres with soil Cd fractions (|*r*| > 0.6, *P* < 0.05). **(B)** Mantel test of metabolites significantly enriched in the LAR and HAR rhizospheres with soil Cd fractions (|*r*| > 0.6, *P* < 0.05). **(C)** Correlation network between metabolites significantly enriched in the LAR rhizosphere and differential bacterial taxa (|*r*| > 0.6, *P* < 0.05). **(D)** Correlation network between metabolites significantly enriched in the HAR rhizosphere and differential bacterial taxa (|*r*| > 0.6, *P* < 0.05). **(E, F)** Partial Least Squares Path Modeling (PLS-PM) illustrating the main pathways through which significantly enriched metabolites affect rice Cd accumulation in LAR and HAR rhizosphere soils, respectively. Rhizospheric metabolites and Rhizospheric microorganisms represent the metabolites and microbes significantly enriched in LAR or HAR rhizosphere soils; Bioavailable Cd content includes ESC-Cd and CAB-Cd; Inert Cd content includes FMO-Cd, OM-Cd, and RES-Cd. **(G, H)** Standardized total effects of metabolites, microbes, bioavailable Cd, inert Cd, and root Cd content on each process in LAR **(G)** or HAR **(H)** rhizosphere soils, as estimated by PLS-PM. The total effects represent the sum of direct and indirect pathways linking each predictor to the response variable. A longer bar indicates a stronger total modeled influence of the predictor on the respective response variable. For example, “A longer bar for ‘Metabolites' on ‘Inert Cd' indicates metabolites have the strongest total modeled influence on promoting inert Cd formation.”

To further elucidate the interactions among metabolites, microbial communities, and Cd speciation in the rhizospheres of rice cultivars with different Cd accumulation capacities, bacteria and metabolites that were significantly enriched in the LAR and HAR rhizosphere soils were analyzed using Mantel tests and Pearson correlation models (*P* < 0.05) to examine their associations with soil Cd speciation. The results indicated that bacteria (Figure 6A) and metabolites (Figure 6B) significantly enriched in the LAR rhizosphere were strongly correlated with ESC-Cd and RES-Cd, whereas those enriched in the HAR rhizosphere were correlated mainly with RES-Cd. These findings suggest that variations in soil bacterial communities and metabolites may influence Cd speciation. Pearson correlation analysis further revealed that the bacterial taxa (Supplementary Tables S12, S13) and metabolite classes (Supplementary Tables S14, S15) are associated with Cd fractions in the LAR and HAR rhizospheres. Metabolites significantly associated with Cd fractions in the LAR and HAR rhizospheres were classified (Supplementary Tables S15, S16), and co-occurrence networks were constructed to visualize correlations between Cd-associated bacteria and metabolites (|*r*| > 0.6, *P* < 0.05). In LAR rhizospheres (Figure 5C), enriched bacteria were significantly associated with lipids and lipid-like molecules, fatty acids, organic acids and derivatives, benzenoids, organoheterocyclic compounds, shikimates and phenylpropanoids, phenylpropanoids and polyketides, polyketides, lignans and neolignans, carbohydrates, alkaloids, terpenoids, and organic nitrogen compounds (Supplementary Table S17). In HAR rhizospheres (Figure 6D), enriched bacteria were correlated mainly with shikimates and phenylpropanoids, lipids and lipid-like molecules, terpenoids, fatty acids, and organoheterocyclic compounds (Supplementary Table S18). These results suggest that the interactions between characteristic metabolites and bacterial populations in LAR and HAR rhizospheres create distinct rhizosphere microenvironments, thereby regulating Cd speciation.

Partial least squares path modeling (PLS-PM) was applied to quantify the relationships among rhizosphere soil metabolites, bacterial communities, Cd speciation, and rice Cd accumulation (Figures 6E, F). In LAR rhizospheres (Figure 6E), metabolites directly positively affected the bacterial and inert Cd contents (*P* < 0.05), whereas soil bacteria directly influenced the bioavailable and inert Cd contents, with positive and negative effects, respectively (*P* < 0.05). In HAR rhizospheres (Figure 6F), metabolites directly promoted bacterial abundance (*P* < 0.05) but negatively affected inert Cd fractions (*P* < 0.05); bacterial influences on Cd fractions were not significant (*P* > 0.05). In terms of standardized total effects, rhizosphere metabolites contributed the most to Cd speciation, followed by rhizosphere bacterial communities (Figures 6G, H).

### 3.6 Effects of characteristic LAR metabolites on soil Cd speciation

Based on the collective results of these analyses, we hypothesized that soil metabolites influence Cd speciation by modulating associated microbial communities. Accordingly, differentially enriched metabolites in the LAR rhizosphere were selected on the basis of their VIP values and fold changes (Supplementary Table S19). Selection also considered compound safety and commercial availability for subsequent experimental validation. Ultimately, 23 metabolites were chosen, including sinapic acid, isosteviol, biotin, 6,7-diethoxy-4-methylcoumarin, retinoic acid, dehydroabietic acid, lumichrome, hypoxanthin, allopurinol, hecogenin, imatinib, totarol, hydroxyisocaproic acid, 2-ethyl-2-hydroxybutyric acid, 2-hydroxyhexanoic acid, isoferulic acid, ferulate, arachidonic sulfonic acid, pentadecana, undecanoic acid, homoveratric acid, hydroxytyrosol acetate and hydroferulic acid. These compounds were combined into a metabolite mixture to test the hypothesis that rhizosphere metabolites drive the transformation of soil Cd speciation and reduce Cd bioavailability. The 23 metabolites represented diverse chemical classes, including shikimates and phenylpropanoids, terpenoids, phenylpropanoids and polyketides, lipids and lipid-like molecules, alkaloids, organoheterocyclic compounds, fatty acids, organic acids and derivatives, and other compounds.

**Figure 7:**
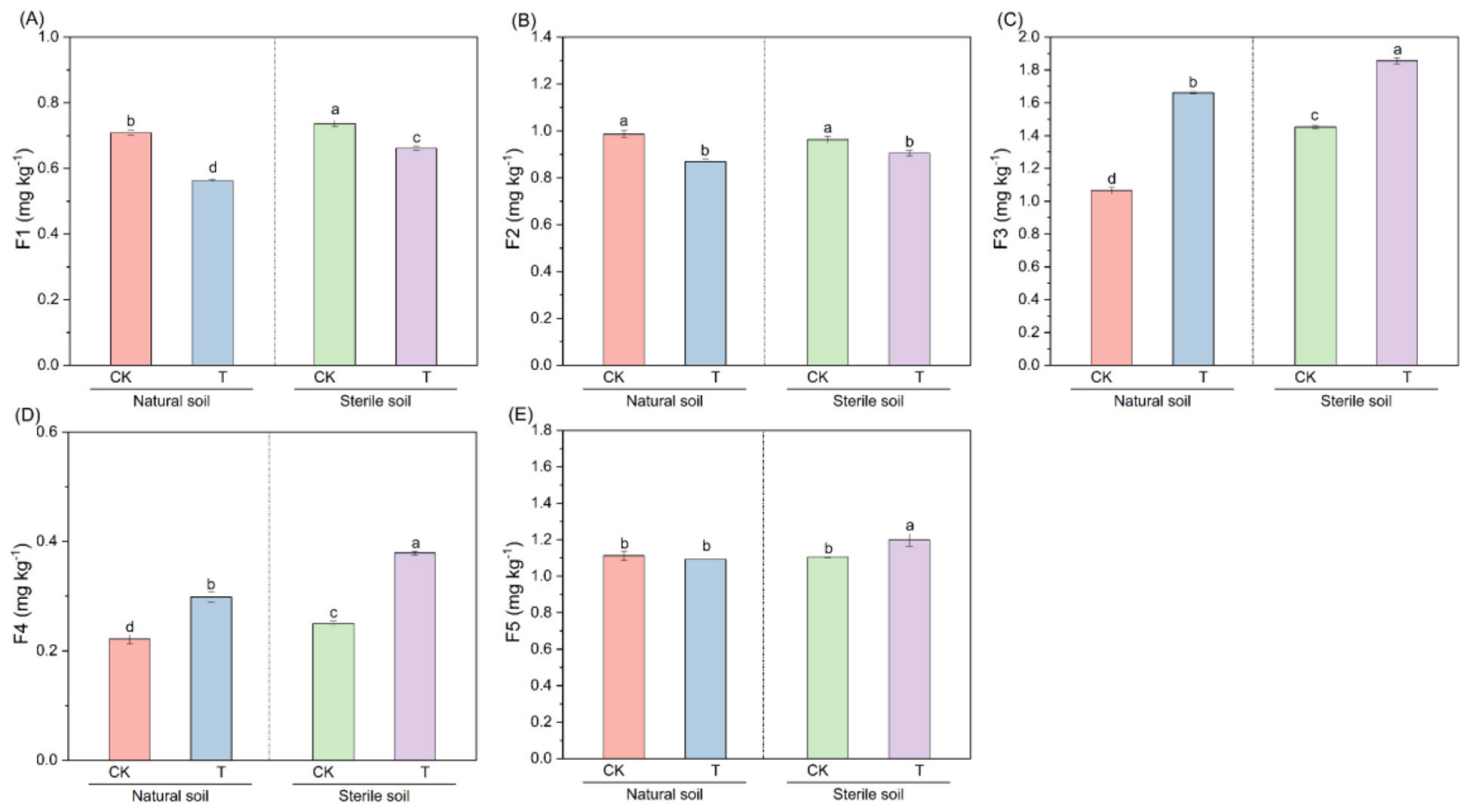
Rhizosphere metabolites drive the transformation of soil Cd speciation. **(A–E)** Contents of F1 (ESC-Cd), F2 (CAB-Cd), F3 (FMO-Cd), F4 (OM-Cd), and F5 (RES-Cd) after the addition of the metabolite mixture.

A soil incubation experiment was conducted to verify the effects of rhizosphere metabolites on Cd speciation (Figures 7A–E). The results revealed that the addition of rhizosphere metabolites to natural soil led to Cd immobilization, as indicated by significant decreases in ESC-Cd and CAB-Cd and increases in iron-manganese oxide-bound cadmium (FMO-Cd) and OM-Cd (*P* < 0.05). The addition of the same metabolites to sterilized soil also induced Cd immobilization, although the effect was markedly weaker than was the case in natural soil. For example, under treatment T, ESC-Cd decreased by 20.6% in natural soil, whereas in sterilized soil, it decreased by only 10.2% (Figure 7A); likewise, FMO-Cd increased by 56.0% in natural soil but only by 27.8% in sterilized soil (Figure 7C). These results indicate that rhizosphere metabolites can directly participate in Cd immobilization and exert indirect effects through microbial activity.

## 4 Discussion

The fact that plant genotypes and rhizosphere soil characteristics jointly influence Cd bioavailability has been well established (Hou et al., 2018). In this study, varietal screening revealed that, compared with HAR, LAR resulted in significantly lower Cd concentrations in both roots and grains. Moreover, the bioavailable Cd speciation (ESC-Cd and CAB-Cd) in the rhizosphere were markedly lower, while those in the inert Cd fractions (OM-Cd and RES-Cd) were significantly greater (Figure 2). These findings indicate that Cd speciation in the rhizosphere is a critical determinant of differences in Cd accumulation among rice cultivars. Rhizosphere metabolites and microorganisms are known to profoundly influence Cd bioavailability (Lu et al., 2023; Wang et al., 2023), a conclusion further supported by our PLS-PM analysis. Integrated metagenomic and metabolomic analyses revealed distinct metabolite profiles differentiating the LAR and HAR groups. A characteristic metabolite set comprising 23 metabolites significantly enriched in the LAR rhizosphere was identified and, more importantly, experimentally validated through soil incubation experiments, providing direct causal evidence that this metabolite consortium substantially reduces soil Cd bioavailability (Figure 6). Collectively, the results of this study provide compelling evidence that rice cultivars with contrasting Cd accumulation capacities harbor distinct rhizosphere microecosystems and establish an adaptive mechanism through which the rhizosphere metabolites of LAR drive Cd speciation transformation.

### 4.1 Rhizosphere metabolomic profiles of different rice cultivars

Rhizosphere metabolites include both root exudates and microbial metabolic products. Although several studies have investigated the metabolite compositions of rhizosphere soils of rice under Cd stress, most investigations have been restricted to only one or two cultivars (Liu et al., 2021; Wang et al., 2024). However, studies elucidating generalizable patterns of rhizosphere metabolite responses to Cd contamination across broader genetic backgrounds remain scarce. In this study, comparative metabolomic analysis between LAR (four cultivars) and HAR (three cultivars) revealed distinct metabolite variations corresponding to differing Cd accumulation capacities. Specifically, the relative abundances of organic acids and derivatives, shikimates and phenylpropanoids, benzenoids, terpenoids, and fatty acids were greater in the LAR rhizospheres than in the HAR rhizospheres, which is consistent with the findings reported by Wang et al. (2025)). Overall, 129 metabolites were significantly upregulated and 163 were significantly downregulated in the LAR rhizosphere relative to those in the HAR rhizosphere. Further analysis revealed that the characteristic LAR metabolites are predominantly fatty acids, accounting for 41% of the total relative abundance, whereas the characteristic HAR metabolites show a more uniform composition (Supplementary Table S6). These results suggest that LAR may suppress systemic metabolic diversification to promote the development of functionally specialized metabolites. Previous studies have reported that Cd stress increases the exudation of amino acids—including histidine, methionine, phenylalanine, and lysine—from the roots of both high accumulating (Lu527-8) and conventional (Lu527-4) rice lines, with the presence of histidine strongly positively correlated with Cd levels (Fu et al., 2019). Similarly, Wang et al. (2025)) demonstrated that characteristic LAR metabolites (e.g., hexaconazole and ribose 1,5-bisphosphate) contribute to complex biochemical and physiological processes that reduce Cd accumulation, whereas metabolites enriched in the HAR rhizosphere (e.g., quercetin 3-arabinoside and 5-dehydroavenasterol) facilitate Cd uptake.

### 4.2 Rhizosphere microbial community characteristics of different rice cultivars

Rhizosphere microorganisms play pivotal roles in the mobilization and immobilization of soil Cd (Song et al., 2018; Zhao et al., 2023). Hou et al. (2018)) demonstrated that rice cultivars differing in their Cd uptake capacity harbor distinct rhizosphere microbial communities. In this study, both the species and functional richness (Chao1 index) of the LAR rhizosphere were significantly lower than those of the HAR rhizosphere (*P* < 0.05), and the Shannon diversity index also tended to be lower. These results are consistent with those reported by Wang et al. (2025)), who reported that the LAR rhizosphere microbial community generally exhibited relatively low richness and diversity. Network topology analysis further revealed that, compared with HAR, the LAR rhizosphere microbial co-occurrence network exhibited lower density, clustering coefficient, and eigenvector centrality but greater modularity. Similar to the metabolomic profile, distinct microbial co-occurrence patterns were observed between LAR and HAR under Cd stress. Specifically, HAR—characterized by greater Cd accumulation—tend to harbor a more diverse and functionally versatile microbial network adapted to Cd-contaminated environments, whereas LAR may foster a functionally specialized microbial community that facilitates Cd immobilization at the cost of reduced community and functional diversities. For instance, the biosynthesis of various alkaloids is significantly enriched in the LAR rhizosphere, and alkaloid formation is known to facilitate Cd^2^^+^ chelation and precipitation (Qi et al., 2023). Moreover, *Deltaproteobacteria bacterium* and *Candidatus Sulfobium mesophilum*—both significantly enriched in the LAR rhizosphere—have been reported to play key roles in alleviating Cd stress in crops (Chen et al., 2024; Zecchin et al., 2018). Similarly, Wang R. et al. (2020)) demonstrated that the nonaccumulating ecotype (NAE) of *Sedum alfredii* developed a less phylogenetically diverse, yet functionally specialized, microbial community under heavy metal stress, effectively restricting heavy metal uptake by plants. Collectively, these findings suggest that LAR cultivars shape a simplified yet functionally targeted rhizosphere microbiome that facilitates Cd immobilization, whereas HAR cultivars maintain a more complex and adaptive microbial ecosystem that enhances Cd mobilization and uptake. However, the decline of biodiversity may weaken the ecosystem functional redundancy and reduce the resistance and resilience of the community to environmental disturbances (such as drought, temperature fluctuations and pathogen invasion), thereby potentially exerting negative effects on crop yield (Bardgett and Caruso, 2020; Biggs et al., 2020). Yang et al. (2025)) also demonstrated that the loss of microbial diversity reduces both general soil functions (e.g., C and N mineralization, soil respiration, biomass production) and specific functions (e.g., pollutant degradation, methane metabolism, pathogen control). Accordingly, future studies should implement long-term field monitoring combined with multi-site and multi-temporal sampling to systematically evaluate the dynamics and stability of rhizosphere microbial communities under continuous cultivation of LAR, and to clarify their effects on soil physicochemical properties, Cd speciation transformation, as well as crop yield and quality.

### 4.3 Microbial-driven mechanisms by which rhizosphere metabolites reduce Cd bioavailability

Key rhizosphere metabolites function as both signaling molecules and nutrient sources that mediate microbial colonization processes and activity (Zhao et al., 2024; Fan et al., 2025). The specific microbial responses to these metabolites represent important driving forces underlying rhizosphere microbial community assembly. In this study, distinct differences were observed in the rhizosphere metabolic profiles and bacterial community compositions between the LAR and HAR cultivars. Moreover, variations in Cd speciation within the rhizosphere were significantly correlated with characteristic metabolites and dominant bacterial taxa. PLS-PM analysis further indicated that the characteristic responses in rice with different Cd accumulation capacities drive the assembly of the rhizosphere microbiome, thereby influencing Cd availability.

Certain metabolites can serve as preferred carbon sources, selectively stimulating microbial proliferation (Franzosa et al., 2019). Our results show that characteristic LAR metabolites promote the enrichment of functional bacterial taxa involved in Cd immobilization. *Desulfopila aestuarii* and *Paludibacter* sp. were significantly enriched in LAR rhizospheres; both are sulfate-reducing bacteria (SRB) (Suzuki et al., 2007; Liang et al., 2013), whose activity promotes CdS precipitation, thereby reducing Cd bioavailability. Organic acids and fatty acids serve as primary carbon sources for microorganisms and essential electron donors for microbial sulfate reduction (Behrendt et al., 2024; Ayangbenro et al., 2018). In contrast with HAR, characteristic LAR metabolites were classified mainly as fatty acids and organic acids and derivatives (Supplementary Table S6). Hydroxyisocaproic acid, 2-hydroxyhexanoic acid, and 2-ethyl-2-hydroxybutyric acid were positively correlated with the abundance of *Desulfopila aestuarii*, suggesting that these metabolites may serve as carbon and electron sources for this SRB. Gittel et al. (2010)) reported that *Desulfopila inferna* sp. nov. can utilize organic acids and fatty acids as carbon sources and electron donors to carry out sulfate reduction. Additionally, the abundance of hypoxanthine, a purine alkaloid significantly enriched in the LAR rhizosphere, was strongly positively correlated with that of characteristic LAR bacterial taxa, such as *Desulfopila aestuarii* and *Paludibacter* sp. (Supplementary Table S16). Afridi et al. (2024)) confirmed that hypoxanthine can serve as a carbon source for certain bacteria, supporting its ecological role in selective microbial enrichment. Collectively, these findings suggest that characteristic LAR metabolites facilitate the enrichment and metabolic activity of sulfate-reducing bacteria by providing carbon and electron donors, thereby promoting Cd immobilization.

Conversely, some metabolites exhibit antimicrobial properties that selectively inhibit microbial growth (Franzosa et al., 2019). Antimicrobial metabolites enriched in LAR rhizospheres suppressed the growth of bacteria involved in Cd mobilization. In particular, ferulate, isosteviol, pentadecanal, dehydroabietic acid, and 2-hydroxyhexanoic acid—metabolites significantly enriched in LAR—have been reported to possess potent antimicrobial activities (Ma et al., 2025; Singh et al., 2019; Kim et al., 2019). Integrated metabolomic–metagenomic analyses revealed that these antimicrobial metabolites were positively correlated with the abundance of characteristic LAR bacteria but negatively correlated with the abundance of characteristic HAR bacterial taxa such as *Sulfuriferula* sp. AH1, *Nocardioides deserti*, and *Nocardioides glacieisoli* (Figure 6C). For instance, *Sulfuriferula* sp. *AH* participates in sulfur oxidation (Jones et al., 2017), and its reduced abundance may favor CdS stability, thereby decreasing Cd bioavailability. Furthermore, the suppression of *Nocardioides* (genus), which is associated with Cd tolerance and accumulation in *D. pinnata* (Li et al., 2022), may also contribute to reduced Cd uptake by rice. In summary, LAR rhizosphere metabolites not only promote the growth of Cd-immobilizing bacteria by serving as carbon sources but also selectively inhibit Cd-mobilizing bacteria through antimicrobial metabolites, thereby establishing a rhizosphere microecological mechanism that simultaneously suppresses Cd mobilization while enhancing immobilization.

**Figure 8:**
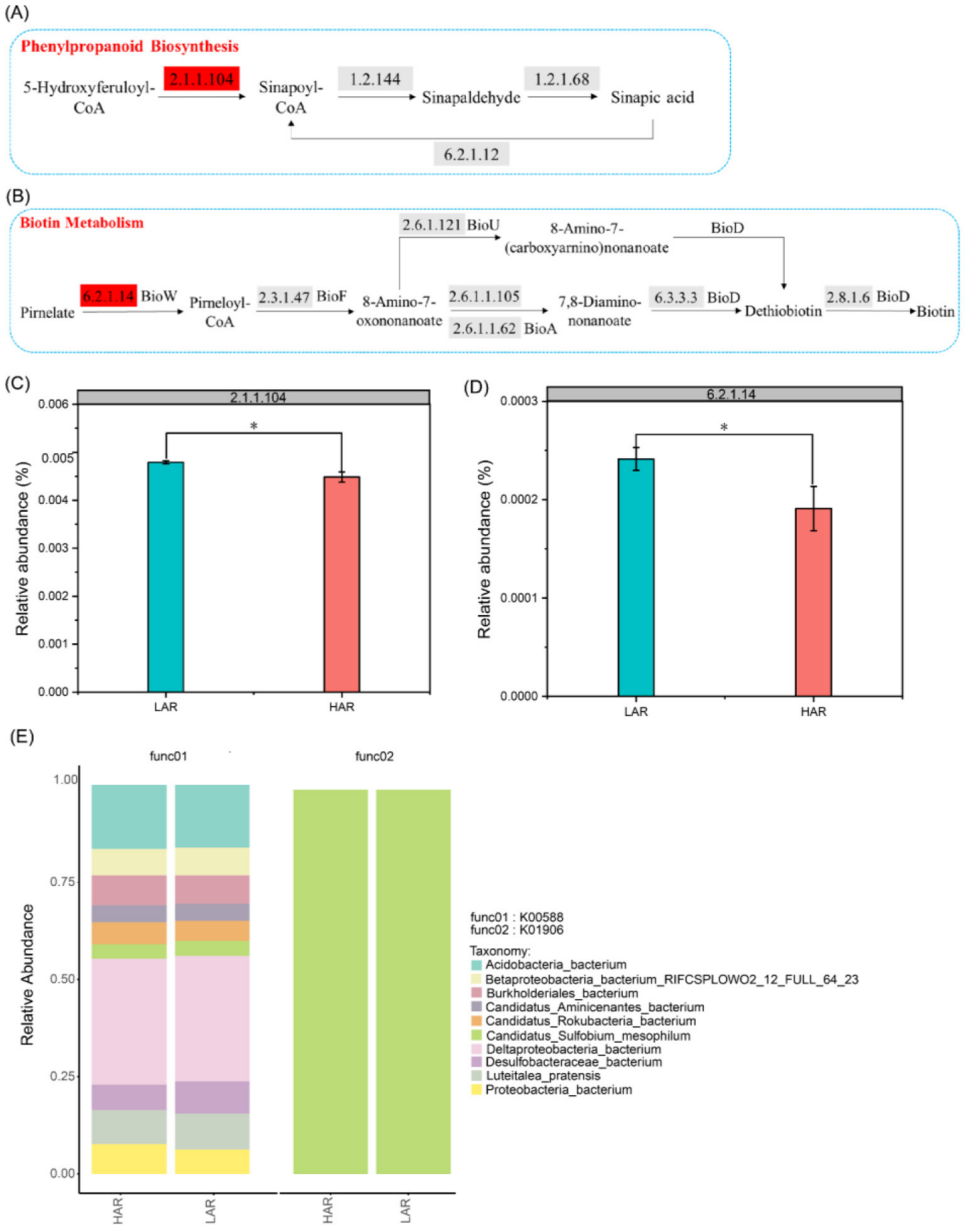
Differentially enriched KEGG pathways revealed by integrated multi-omics analysis. **(A, B)** Differentially enriched KEGG pathways (K00588 and K01906) identified from the metagenomic datasets. **(C, D)** Changes in the gene abundance of enzymes involved in the K00588 and K01906 metabolic pathways. **(E)** Column chart of microbial contribution at the species level based on K00588 and K01906 functional pathways.

Moreover, characteristic LAR metabolites recruit bacterial taxa to participate in specific rhizosphere metabolic pathways. Multiomics integration revealed that (Figures 8A–E), compared with the HAR, the LAR presented significantly greater abundances of microbial genes encoding enzymes involved in phenylpropanoid biosynthesis (K00588) and biotin metabolism (K01906). These included genes encoding caffeoyl-CoA O-methyltransferase [EC: 2.1.1.104] and 6-carboxyhexanoate–CoA ligase [EC: 6.2.1.14]. Species contribution analysis (Figure 8E) revealed that *Acidobacteria bacterium, Candidatus Sulfobium mesophilum*, and *Deltaproteobacteria bacterium* were the primary contributors to caffeoyl-CoA O-methyltransferase, whereas *Candidatus Sulfobium mesophilum* contributed to 6-carboxyhexanoate–CoA ligase. Notably, both *Candidatus Sulfobium mesophilum* and *Deltaproteobacteria bacterium* were significantly enriched in the LAR rhizospheres. These findings indicate that characteristic LAR metabolites can reshape the abundance of specific microbes and the associated functional genes, thereby regulating rhizosphere metabolic pathways. Furthermore, the biosynthesis of sinapic acid and biotin has been reported to decrease rhizospheric Cd bioavailability and inhibit Cd accumulation in rice (Kovácik and Klejdus, 2008; Chen et al., 2025). These results highlight the intricate interactions between metabolites and microbes within the rice rhizosphere microecosystem and underscore a key regulatory process in which rhizosphere metabolites modulate specific microbial metabolic pathways, thereby ultimately influencing Cd bioavailability.

Rhizosphere metabolites can also directly interact with heavy metal ions, thereby mitigating their toxicity to plants (Zadel et al., 2022). For example, biotin and 6,7-diethoxy-4-methylcoumarin, which are significantly enriched in the LAR rhizosphere, can directly chelate Cd^2^^+^, thereby reducing Cd bioavailability (Zhang et al., 2006; Szwaczko et al., 2025). In this study, by adding a characteristic metabolite consortium derived from LAR rhizosphere to both sterilized and natural soils, we further distinguished the direct and indirect mechanisms by which metabolites regulate Cd speciation transformation. The results showed that in sterilized soil, ESC-Cd decreased by 10.2%, which can be attributed to the direct chemical immobilization of Cd by metabolites through mechanisms such as chelation and complexation. In natural soil, ESC-Cd decreased by 20.6%, a reduction significantly greater than that observed under sterilized conditions. The difference between the two soil conditions (10.4%) can be regarded as the indirect effect of metabolites by regulating the rhizosphere microbial community. This result clearly showed that about 50.5% (10.4/20.6 × 100%) of the contribution of rhizosphere metabolites to Cd immobilization came from the indirect pathway mediated by microorganisms, and the remaining 49.5% came from the direct chemical action of metabolites themselves. Similarly, FMO-Cd increased by 27.8 and 56.0% in sterilized and natural soils, respectively, and the difference (28.2%) further confirmed the key role of microorganisms in driving Cd speciation transformation. In summary, through sterilization-based comparative experiments, this study quantitatively disentangled the relative contributions of direct and indirect effects of rhizosphere metabolites in Cd immobilization, revealing a synergistic mechanism by which both pathways jointly drive Cd speciation transformation. However, this study did not include direct measurements of Cd accumulation in rice and therefore provides limited evidence regarding the direct influence of characteristic LAR metabolites on Cd uptake in rice. Future studies involving rice cultivation experiments should be conducted to validate these effects.

## 5 Conclusions

This study demonstrates that rice cultivars with different Cd accumulation capacities can regulate soil Cd bioavailability by establishing distinct rhizosphere microecological systems. Through correlation analyses and experimental validation, this study provides direct evidence for the role of rhizosphere LAR metabolites in soil Cd immobilization and reveals a novel mechanism whereby Cd immobilization is jointly driven by direct chemical interactions of metabolites and indirect microbially mediated processes. These findings highlight that the rhizosphere microecosystem is one of the key determinants of cultivar-dependent differences in Cd accumulation and provide a theoretical basis for the selection and breeding of LAR using rhizosphere-based regulatory strategies. Future work should focus on optimizing combinations of key metabolites and evaluating their long-term effectiveness under field conditions to strengthen rhizosphere ecological barriers against Cd contamination in rice.

## Data Availability

Sequence data that support the findings of this study have been deposited in the NCBI Sequence Read Archive (SRA) database under BioProject ID PRJNA1366918.
