# Supplementary material for: Characteristic rhizosphere metabolites of low-cadmium-accumulating rice drive microbially induced soil cadmium speciation transformation
Source: Front Microbiol. 2026 Apr 22;17:1832350. doi: 10.3389/fmicb.2026.1832350 (PMC13144023; doi:10.3389/fmicb.2026.1832350)
Supplement: Supplementary file 1 [file Table_1.docx]

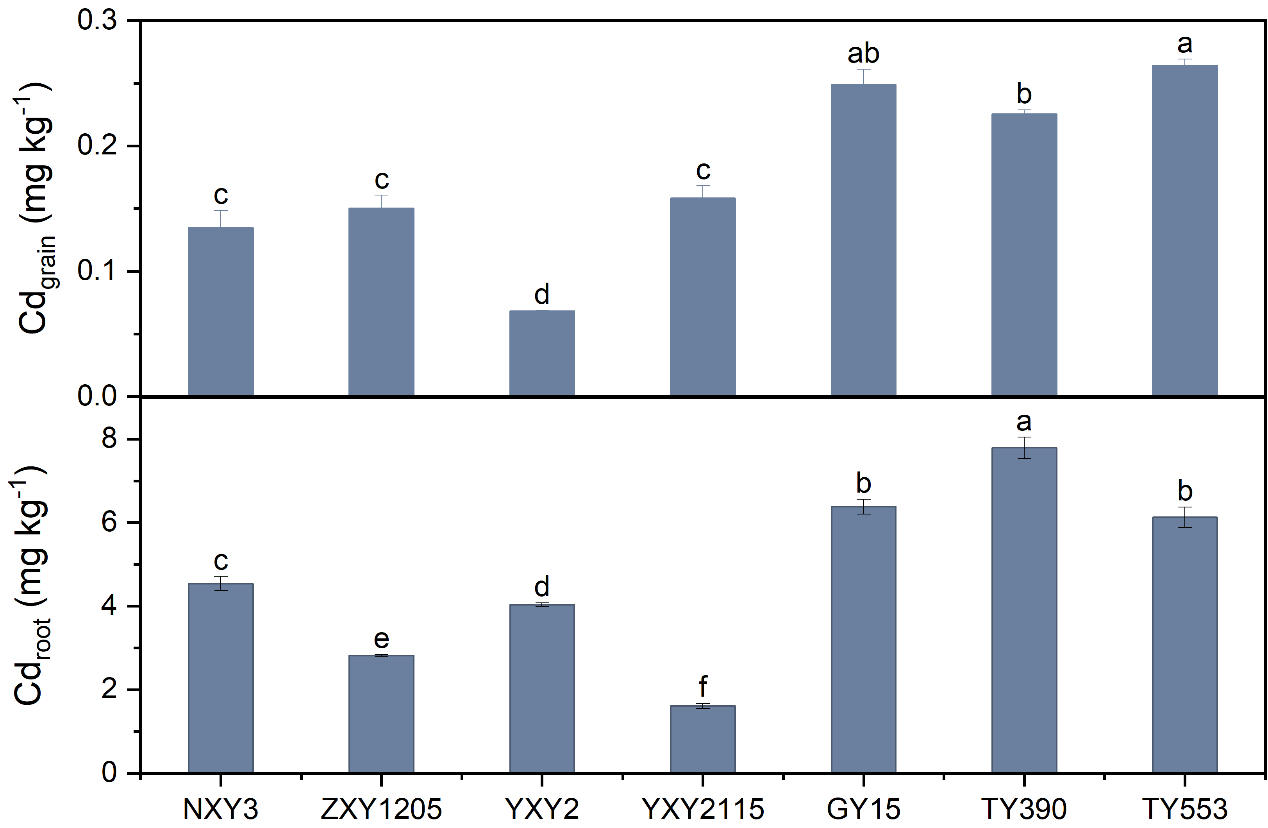


Fig. S1. Cd content in grain and root of low Cd and high Cd accumulation rice


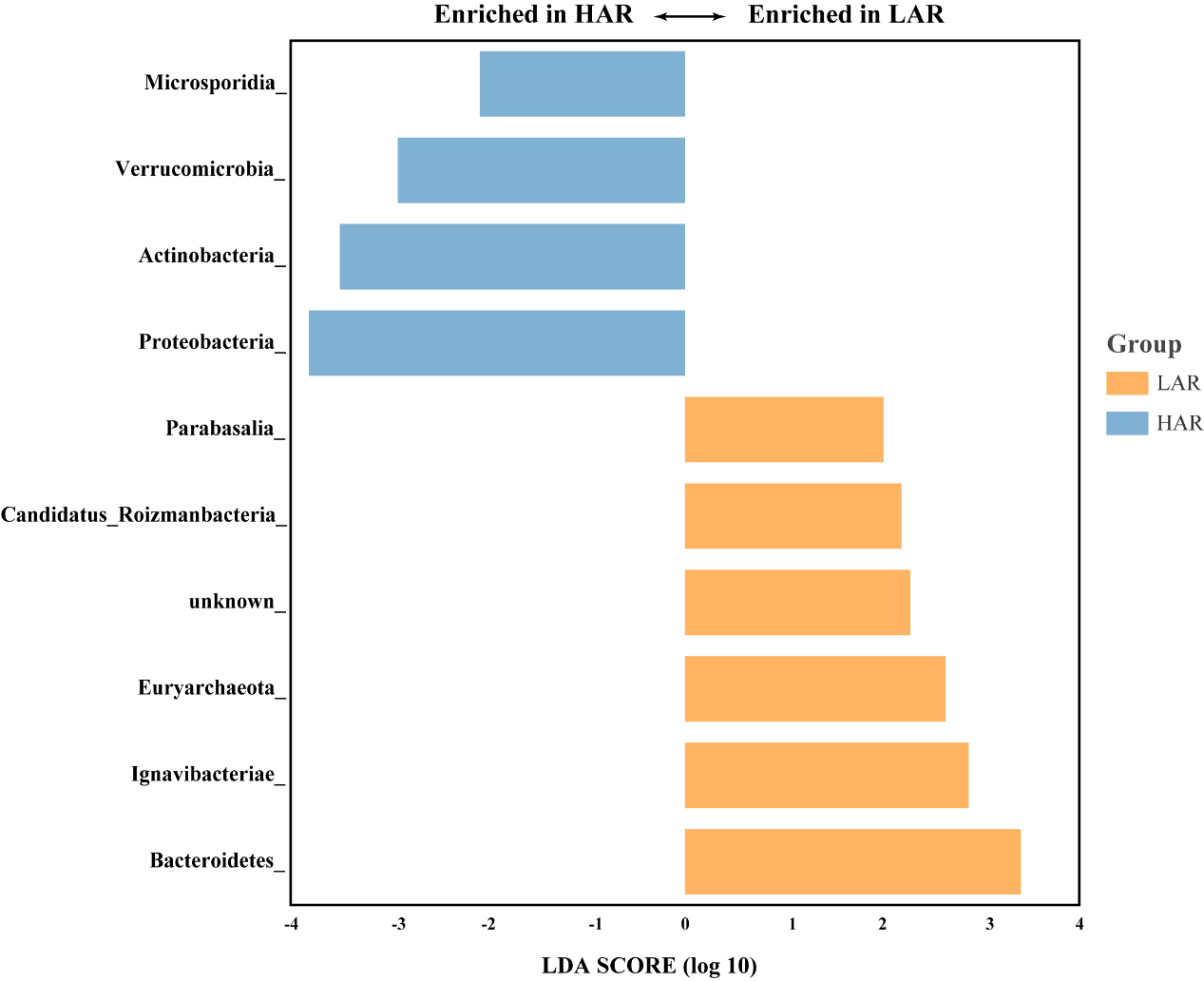
Fig. S2. Differences in rhizosphere bacteria between LAR and HAR at the phylum level.


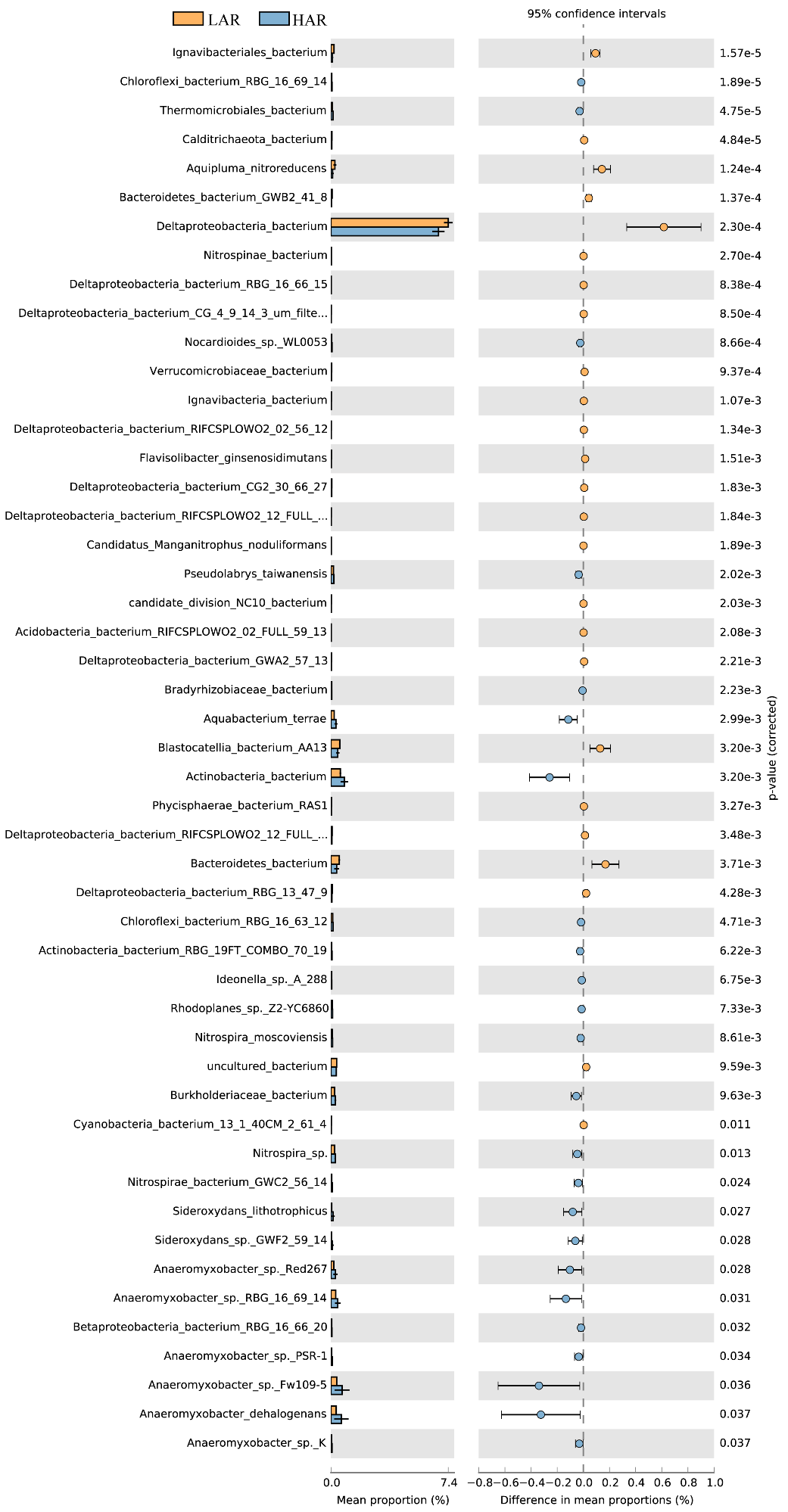
Fig. S3. The main bacteria (species level) differs significantly between LAR and HAR based on Kruskal–Wallis test.
